# Supplementary material for: Temperamental Change in Adolescence and Its Predictive Role on Anxious Symptomatology
Source: Behav Sci (Basel). 2022 Jun 16;12(6):194. doi: 10.3390/bs12060194 (PMC9219936; doi:10.3390/bs12060194)
Supplement: Supplementary file 1 [file behavsci-12-00194-s001.zip › behavsci-1761972-supplementary.pdf]

behavioral sciences

MDPI

Article

Temperamental Change in Adolescence and its Predictive Role on Anxious Symptomatology

Supplementary Materials: The following are available online at www.mdpi.com/xxx/s1, Table S1: Parameters on the multigroup growth model (configural model).

	Boys				Girls			
	Estimate	*SE*	*z*	*p*	Estimate	*SE*	*Z*	*p*
Regressor on Anxiety score								
BIS latent intercept	2.68	0.66	4.09	0.00	3.15	0.64	4.95	0.00
AC latent intercept	-4.22	3.15	-1.34	0.18	-7.10	5.45	-1.30	0.19
BIS latent slope	3.45	3.57	0.97	0.34	10.34	3.41	3.03	0.00
AC latent slope	-28.01	58.84	-0.48	0.63	-1.17	32.96	-0.04	0.97
Covariance								
BIS latent intercept ~~								
BIS latent slope	-0.79	0.36	-2.17	0.03	-0.56	0.32	-1.75	0.08
AC latent intercept	-0.34	0.19	-1.76	0.08	-0.66	0.13	-5.04	0.00
AC latent slope	-0.02	0.06	-0.30	0.76	0.06	0.05	1.35	0.18
BIS latent slope ~~								
AC latent intercept	0.08	0.07	1.11	0.27	0.09	0.05	1.82	0.07
AC latent slope	-0.03	0.02	-1.30	0.19	-0.04	0.02	-2.35	0.02
AC latent intercept ~~								
AC latent slope	-0.01	0.02	-0.79	0.43	0.00	0.01	0.26	0.79
Intercept								
Anxiety score	27.46	15.77	1.74	0.08	41.90	29.67	1.41	0.16
BIS latent intercept	5.88	0.30	19.38	0.00	7.29	0.24	30.12	0.00
BIS latent slope	-0.26	0.11	-2.29	0.02	-0.49	0.08	-5.81	0.00
AC latent intercept	4.47	0.06	78.63	0.00	4.53	0.04	116.58	0.00
AC latent slope	0.00	0.02	-0.08	0.93	0.01	0.01	0.50	0.62
Variance								
BIS T1	1.95	0.86	2.26	0.02	3.35	0.72	4.64	0.00
BIS T2	4.16	0.70	5.90	0.00	3.56	0.62	5.73	0.00
BIS T3	4.77	1.40	3.40	0.00	3.75	0.52	7.17	0.00
BIS T4	2.30	0.83	2.78	0.01	3.03	0.89	3.41	0.00
BIS T1	0.21	0.05	4.39	0.00	0.15	0.02	6.28	0.00
BIS T2	0.22	0.04	5.35	0.00	0.12	0.02	6.85	0.00
BIS T3	0.10	0.02	5.12	0.00	0.13	0.02	6.56	0.00
BIS T4	0.11	0.03	4.10	0.00	0.11	0.03	4.48	0.00
Anxiety score	127.23	22.76	5.59	0.00	132.40	18.72	7.07	0.00
BIS latent intercept	7.46	1.09	6.85	0.00	8.61	1.05	8.21	0.00
BIS latent slope	0.65	0.20	3.32	0.00	0.66	0.18	3.62	0.00
AC latent intercept	0.21	0.05	4.11	0.00	0.19	0.03	7.13	0.00
AC latent slope	0.01	0.01	0.87	0.38	0.01	0.01	1.89	0.06

Note. Observable variable loadings and intercepts are fixed due to specification of the latent growth model.

BIS = Behavioral inhibition system. AC = Attentional control. SE = Standard error of the estimate.
